# The Gut-Brain Connection: A Case Report on Depression in Ulcerative Colitis and Novel Treatment Approach

**DOI:** 10.7759/cureus.48462

**Published:** 2023-11-07

**Authors:** Aditi Sharma, Vinod Sharma

**Affiliations:** 1 Psychiatry, The Wright Center for Graduate Medical Education, Scranton, USA

**Keywords:** quality of life, cognitive-behavioral therapy, multidisciplinary care, integrated care, inflammatory bowel disease, comorbidity, depression, ulcerative colitis

## Abstract

Ulcerative colitis (UC) is a chronic inflammatory bowel disease characterized by mucosal inflammation in the colon, leading to a range of gastrointestinal symptoms. Emerging research has shown significant comorbidity between UC and depression, with a bidirectional relationship that further complicates disease management and patient well-being. Depression is more prevalent among individuals with UC, affecting 27% of them, compared to the general population's 12%. Factors contributing to depression in UC patients include the chronic nature of the disease, frequent hospitalizations, unpredictable flares, and associated physical symptoms such as pain, fatigue, and altered body image. Depression, in turn, can exacerbate UC symptoms, leading to a vicious cycle of disease progression and emotional distress. A new treatment approach has surfaced as a solution to the challenge of providing effective care to patients with UC who also suffer from depression. This approach involves a collaborative effort by a multidisciplinary team consisting of gastroenterologists, psychiatrists, psychologists, and dietitians, who work together to provide comprehensive care to UC patients with depression. This case report discussed the psychiatric presentation and management of a 75-year-old male who developed severe depression following his diagnosis of UC. This patient demonstrated significant improvement in depressive symptoms with the integrated care approach. This case highlights the importance of recognizing and addressing the psychological impact of chronic inflammatory conditions such as UC and the potential benefits of integrating psychopharmacological interventions into the treatment plan. Further research is essential to establish the effectiveness of this approach and refine its implementation in clinical practice.

## Introduction

Ulcerative colitis (UC) is a chronic inflammatory bowel disease (IBD) that not only affects physical health but can also considerably impact mental well-being [1]. According to studies, depression is present in 27% of IBD patients as compared to 12% in controls without IBD [1,2]. UC presents with higher depression rates as compared to any other chronic illness [1,2]. As reported in a recent meta-analysis, the prevalence of depression and anxiety was 23% and 32.6% in patients with UC, respectively [1]. Moreover, 41.3% of patients with active UC had depression [1]. IBD and psychiatric comorbidity have been found to have a bidirectional relationship [1-3]. Depression has been highlighted as a risk factor for clinical recurrence of UC and has a detrimental effect on disease progression [4]. Clinical guidelines emphasize treatment with antidepressants in IBD patients as it will not only improve mood and anxiety but also have anti-inflammatory properties as well [2]. This indicates that relief of depression and anxiety potentially affects gut health which is referred to as the “brain-gut axis” [3,5]. Although there is still much to learn about the psychological consequences of UC and its treatment, we present a case that highlights the interplay between physical illness and mental health. We discuss the emergence of severe depression in a patient with UC and a multidisciplinary approach to the treatment. 

## Case presentation

A 75-year-old male with no prior history of psychiatric illness was referred to our psychiatric clinic due to persistent low mood, anhedonia, disrupted sleep, apathy, personality change, and diminished interest in activities of daily living. These symptoms emerged two months after he was diagnosed with UC. He was on adalimumab 40 mg subcutaneously every other week and a 4-week course of oral prednisone 40 mg daily. Upon psychiatric evaluation, the patient met the diagnostic criteria for major depressive disorder (MDD) based on the Diagnostic and Statistical Manual-5 criteria. The Patient Health Questionnaire (PHQ-9) score was 25 indicating severe depression, significantly impairing his quality of life, and appeared to be associated with the recent diagnosis of UC. A thorough neurological workup was done, and organic neurological etiology was ruled out. Various antidepressants were tried including bupropion, sertraline, duloxetine, and amitriptyline, augmented with aripiprazole but with minimal to no response. Augmentation therapy with olanzapine, an atypical antipsychotic with mood-stabilizing properties, was chosen due to its potential to address both depressive symptoms and sleep disturbances that showed improvement in his symptoms. A multidisciplinary approach was followed for his treatment that incorporated combining gastroenterological management, cognitive behavioral therapy (CBT), dietary adjustments, and an individualized medication plan. 

Treatment and progress

Gastrointestinal Management

The UC was managed with a four-week course of corticosteroid therapy and adalimumab to achieve clinical remission of the disease. 

Psychiatric Intervention

The patient was started on a combination of bupropion and olanzapine for his psychiatric symptoms. Olanzapine 10 mg at bedtime was used as an augmenting agent with bupropion extended release 450 mg daily. He was enrolled in CBT and mindfulness-based stress reduction programs to address his depressive symptoms. The patient also received psychoeducation about his physical and mental health. 

Dietary Modification

The patient was referred to a dietitian who recommended a gut-friendly diet rich in fiber and probiotics to ameliorate gastrointestinal symptoms and improve overall well-being. He was initiated on a probiotic regimen containing strains known for their potential benefits in gastrointestinal symptoms and mood regulation. 

After a three-month follow-up, the patient reported improved mood, increased appetite, enhanced sleep quality, and regained interest in his hobbies. The PHQ-9 score decreased significantly from 25 (severe depression) to 6 (mild depression). Furthermore, his gastrointestinal symptoms demonstrated improvement, aligning with his improved mental well-being. Over six months, the patient experienced significant improvement in UC and depressive symptoms, highlighting the potential effectiveness of this novel approach. This case report sheds light on the importance of recognizing and addressing the gut-brain connection in UC and offers valuable insights for clinicians seeking to improve the holistic care of patients with this challenging comorbidity. 

## Discussion

Chronic inflammatory conditions, such as UC, have the potential to trigger or exacerbate depressive episodes [1-3]. Chronic inflammation in the gut can lead to the release of proinflammatory cytokines. The cytokines can signal the brain through the gut-brain axis, leading to a state of low-grade systemic inflammation. Inflammation can then trigger depression and in turn, depression can increase inflammation by releasing IL-1, IL-6, and IL-12 and tumor necrosis factor (TNF) leading to exacerbation of the UC [3,4]. This heightened inflammation is associated with deteriorating gastrointestinal symptoms and the emergence of depressive symptoms [4]. The intricate interplay between systemic inflammation, neurobiological factors, and psychosocial stressors can contribute to the development of depression [5].

Additionally, though essential for disease management, treatment modalities used for UC can precipitate anxiety and depression [6]. Corticosteroids and adalimumab are known to increase the rates of depression [4,6]. According to the studies, the link between UC and depression is bidirectional [3,4]. The gut-brain axis is a complex and bidirectional communication system that connects the central nervous system (CNS), which includes the brain and spinal cord, with the enteric nervous system (ENS) in the gut [3]. This network involves various components, including neural, hormonal, and immunological pathways, and plays a crucial role not only in the regulation of various bodily functions, including digestion, and metabolism, but also mental health [3,4]. The connection between the gut-brain axis and the emergence of depression in UC patients can be explained as follows:

Altered gut microbiota and dysregulated gut-brain signaling

The gut microbiome is a diverse collection of microorganisms, including bacteria, viruses, protozoa, and fungi, and their genetic material residing in the gastrointestinal tract. In a healthy state, these microbes work in harmony with the gut, supporting intestinal health, energy production, and immune system function [1]. UC often results in imbalances in the gut microbiota. This dysbiosis can affect the composition of beneficial and harmful bacteria in the gut [1,7]. An altered gut microbiota is increasingly recognized for its potential role in mood regulation and depressive disorders [7]. The gut and brain communicate through the vagus nerve, hormones, and neurotransmitters and any disruptions may contribute to mood disturbances [3-6]. 

Gut permeability (leaky gut)

UC can compromise the integrity of the gut lining, leading to increased gut permeability, also known as "leaky gut." This can allow the translocation of bacterial toxins from the gut into the bloodstream, which may trigger an immune response leading to systemic inflammation and, potentially, depressive symptoms [4,6]. 

Genetic predisposition and lifestyle factors

In individuals predisposed to IBD, such as females, smokers, and those who have experienced early life stress, changes in the gut microbiome occur. These changes lead to exaggerated or adverse immune responses, setting off a cascade of events resulting in a detrimental condition known as "inflammatory depression" [6].

Chronic stress

**Figure 1:**
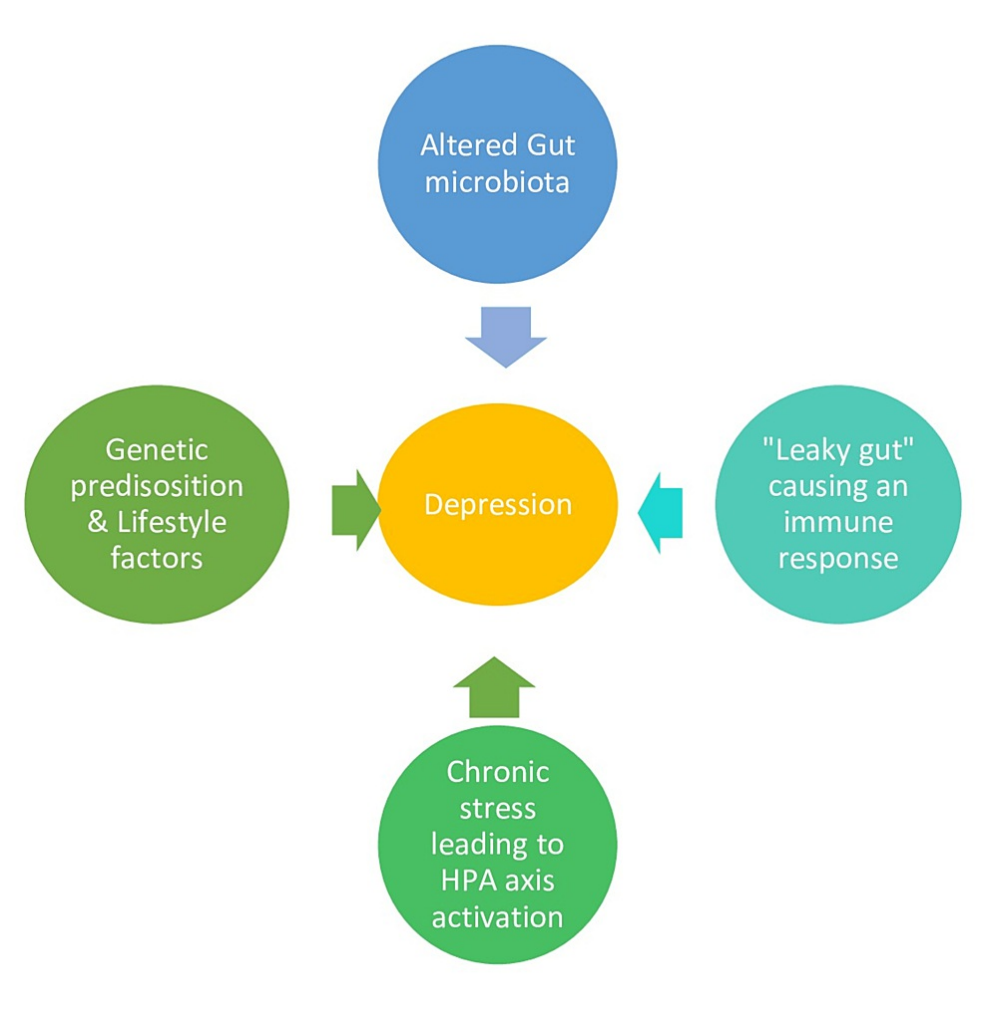
Factors causing depression in Ulcerative colitis HPA: Hypothalamus pituitary adrenal

The impact of UC on a person's daily life, including frequent symptoms, medication management, and social isolation due to the condition, can lead to chronic stress and psychological distress. This leads to overactivity of the hypothalamus pituitary adrenal axis, which contributes to depression [4]. It is important to note that while there is increasing evidence suggesting a connection between UC and depression through the gut-brain axis, the relationship is complex and may vary from one individual to another (Figure 1).

Integrated care models aim to address the physical and emotional aspects of UC simultaneously. These models may include CBT, mindfulness-based interventions, dietary modifications, and medication plans, tailored to the individual patient's needs. The goal is to improve overall quality of life, reduce depressive symptoms, and potentially lead to better disease outcomes in UC patients. In this specific case, trials of several antidepressants like selective serotonin reuptake inhibitors, serotonin and norepinephrine reuptake inhibitors, and tricyclic antidepressants (TCAs) had failed, and a positive response was seen with the combination of olanzapine and bupropion. However, literature shows that TCAs have anti-inflammatory properties by targeting α1-adrenoceptors [8]. Its impact on the inhibitory cytokine IL-10 has been noted, contributing to the suppression of neuroinflammation [8]. Moreover, antidepressants, exert substantial anti-inflammatory effects through a significant reduction in the production of nitric oxide and TNF-α [2,8]. Literature has shown that bupropion can decrease the activity of many diseases where inflammatory mediators are involved in pathophysiology by lowering TNF-α levels and increasing dopamine [8]. Also, the antagonistic role of olanzapine on serotonin and serotonin transporter receptors plays an important role in intestinal motility dysfunction by decreasing the gut motility [4]. Notably, individuals with depression often exhibit abnormally reduced levels of brain-derived neurotrophic factor (BDNF), a vital growth factor essential for brain plasticity, memory function, and the overall well-being of neurons. The consumption of probiotics has been demonstrated to boost the expression of BDNF [7]. In addition, CBT has proven to be highly effective in treating both anxiety and depression [6]. CBT involves a variety of techniques, such as adaptive thinking, problem-solving, exposure, and coping strategies. It is worth noting that both pharmacological and psychological treatments are equally effective and considered first-line treatments for depression in UC [4,6]. By adopting this integrated care model, patients can receive a more holistic and effective approach to their treatment. 

Strength(s) and limitation(s)

The study exhibits several notable strengths as it enables a comprehensive examination of a unique clinical case. It presents an innovative treatment approach and adopts an interdisciplinary perspective that integrates gastroenterology, nutrition, and psychiatry prioritizing patient-centric research. An additional commendable feature is its ability to inspire the formulation of further research hypotheses. It is essential, however, to remain mindful of the inherent limitations associated with case reports, including challenges related to generalizability and establishing causation. Furthermore, the novel treatment approach should be interpreted with prudence until substantiated by more robust empirical evidence. 

## Conclusions

This case report emphasizes the necessity of a comprehensive approach when managing patients with chronic inflammatory diseases, considering the potential for psychiatric comorbidities like depression. Integrating psychiatric assessment and treatment alongside medical management is pivotal for achieving optimal outcomes. Providers should be watchful to differentiate symptom etiology due to MDD or due to underlying medical disorder and treat accordingly. Depression in UC provides a unique chance to understand the interplay between the brain and gut axis. Further research is essential to delineate the intricate relationship between inflammatory conditions and psychiatric well-being and to refine therapeutic strategies. 

## References

[1] Yuan X, Chen B, Duan Z (2021). Depression and anxiety in patients with active ulcerative colitis: crosstalk of gut microbiota, metabolomics and proteomics. Gut Microbes.

[2] Kristensen MS, Kjærulff TM, Ersbøll AK, Green A, Hallas J, Thygesen LC (2019). The influence of antidepressants on the disease course among patients with Crohn’s disease and ulcerative colitis-a Danish nationwide register-based cohort study. Inflamm Bowel Dis.

[3] Keefer L, Kane SV (2017). Considering the bidirectional pathways between depression and IBD: recommendations for comprehensive IBD care. Gastroenterol Hepatol.

[4] Katon W, Ciechanowski P (2002). Impact of major depression on chronic medical illness. J Psychosom Res.

[5] Lesley A. Graff, John R. Walker, Charles N. Bernstein (2009). Depression and anxiety in inflammatory bowel disease: a review of comorbidity and management. Inflamm Bowel Dis.

[6] Moulton C, Pavlidis P, Norton C (2019). Depressive symptoms in inflammatory bowel disease: an extraintestinal manifestation of inflammation?. Clin Exp Immunol.

[7] Wallace CJ, Milev R (2017). The effects of probiotics on depressive symptoms in humans: a systematic review. Ann Gen Psychiatry.

[8] Kane S, Altschuler EL, Kast RE (2003). Crohn's disease remission on bupropion. Gastroenterology.

